# Complete Genome Sequence of Citrobacter freundii Myophage Moogle

**DOI:** 10.1128/genomeA.01426-14

**Published:** 2015-01-29

**Authors:** Quynh T. Nguyen, Adrian J. Luna, Adriana C. Hernandez, Gabriel F. Kuty Everett

**Affiliations:** Center for Phage Technology, Texas A&M University, College Station, Texas, USA

## Abstract

Citrobacter freundii is an opportunistic pathogen that has been linked to nosocomial infections, such as brain abscesses and pneumonia. Further study on phages infecting C. freundii may provide therapeutics for these infections. Here, we announce the complete genome sequence of the FelixO1-like myophage Moogle and describe its features.

## GENOME ANNOUNCEMENT

Citrobacter freundii is a Gram-negative bacterium that has been associated with various nosocomial infections, such as urinary tract infections and neonatal meningitis (1, 2). Antibiotic resistance among C. freundii strains is rising (3), leading to the need for alternative treatments for this potentially deadly opportunistic pathogen. The isolation and characterization of bacteriophages infecting C. freundii, such as myophage Moogle, may lead to such treatments for conditions that can no longer be treated with antibiotics.

Bacteriophage Moogle was isolated from a sewage sample collected in Bryan, TX. Phage DNA was sequenced in an Illumina MiSeq 250-bp paired-end run with a 550-bp insert library at the Genomic Sequencing and Analysis Facility at the University of Texas (Austin, TX). Quality-controlled trimmed reads were assembled to a single contig at 13.62-fold coverage using Velvet version 1.2.10. To confirm the completeness of the contig, reads from both the forward and reverse sequencing reactions were assembled. Furthermore, the contig was confirmed by PCR to be complete. The genes were predicted using GeneMarkS (4) and corrected using the software tools available on the Center for Phage Technology (CPT) Galaxy instance (https://cpt.tamu.edu/galaxy-public/). The morphology of Moogle was determined using transmission electron microscopy performed at the Texas A&M University Microscopy and Imaging Center.

Moogle shares 46.6% sequence identity with the Salmonella phage Felix O1 (accession no. NC_005282), as determined by EMBOSS Stretcher (5, 6). Moogle also shares 48.7 and 48.6% sequence identity with Felix O1-like Escherichia phages EC6 (accession no. JX560968.1) and WV8 (accession no. NC_012749), respectively. The differences between the phages occur largely in the hypothetical conserved genes of unknown function. Moogle has an 87,999-bp genome, with a coding density of 88.1%. As with Felix O1, Moogle has a significantly lower G+C content (39%) than that of its host (51.6%) (7, 8). Twenty-one tRNA genes were identified in Moogle by tRNAscan-SE (9), which is comparable to the 22 tRNA genes identified in Felix O1. Additionally, the transfer-messenger RNA (tmRNA) gene, *ssrA*, was identified using ARAGORN (10). Moogle encodes 7 rho-independent transcriptional terminators compared to the 17 rho-independent terminators encoded by FelixO1 (8). Finally, Moogle contains 2 HNH homing endonucleases compared to the five identified in Felix O1.

Moogle is syntenic with phage Felix O1 and encodes the expected core genes associated with DNA replication, DNA packaging, nucleotide biosynthesis/metabolism, morphogenesis, and lysis. Genes encoding DNA replication proteins include a nuclease, helicase, ligase, and polymerase. The TerL of Moogle shows homology to phages that use headful packaging (11). Several structural genes were identified by homology (tail fiber, baseplate wedge, baseplate assembly protein, tape measure, tape measure chaperone [with translational frameshift], major capsid protein, and prohead protease). Genes encoding lysis proteins include an endolysin (soluble lysozyme), an inner membrane spanin, an outer membrane spanin, and a putative holin (12, 13). As with Felix O1-like phages, these proteins are not present in a lysis cassette but instead are scattered around the genome.

### Nucleotide sequence accession number.

The genome sequence of phage Moogle was deposited in GenBank under the accession no. KM236239.
